# Transposon mutagenesis as an approach to improved understanding of *Borrelia* pathogenesis and biology

**DOI:** 10.3389/fcimb.2014.00063

**Published:** 2014-05-20

**Authors:** Tao Lin, Erin B. Troy, Linden T. Hu, Lihui Gao, Steven J. Norris

**Affiliations:** ^1^Department of Pathology and Laboratory Medicine, University of Texas Medical School at HoustonHouston, TX, USA; ^2^Division of Geographic Medicine and Infectious Diseases, Tufts Medical CenterBoston, MA, USA

**Keywords:** *Borrelia burgdorferi*, Lyme disease, transposon mutagenesis, microbial pathogenesis, bacterial physiology, mouse models

## Abstract

Transposon insertion provides a method for near-random mutation of bacterial genomes, and has been utilized extensively for the study of bacterial pathogenesis and biology. This approach is particularly useful for organisms that are relatively refractory to genetic manipulation, including Lyme disease *Borrelia*. In this review, progress to date in the application of transposon mutagenesis to the study of *Borrelia burgdorferi* is reported. An effective *Himar1*-based transposon vector has been developed and used to acquire a sequence-defined library of nearly 4500 mutants in the infectious, moderately transformable *B. burgdorferi* B31 derivative 5A18NP1. Analysis of these transposon mutants using signature-tagged mutagenesis (STM) and Tn-seq approaches has begun to yield valuable information regarding the genes important in the pathogenesis and biology of this organism.

Lyme disease is a tick-transmitted, multi-stage bacterial infection caused by several species of the genus *Borrelia*, including the primary agents *B. burgdorferi, B. garinii*, and *B. afzelii* (Steere et al., 2004). These spirochetes are maintained in nature through a cycle involving transmission between small mammals and ticks of the *Ixodes ricinus* group. Large mammals such as deer are also required for the feeding of the adult ticks and hence egg production. Humans are accidental hosts that are infected by the bite of *Borrelia*-infested ticks, usually of the nymphal stage. Most infected humans develop mild fever, malaise, and a localized skin lesion called erythema migrans days to weeks after the tick bite, followed by dissemination of propagating organisms through the blood and lymphatics to other tissues. Later manifestations may include neurologic effects, joint involvement, cardiac block, and skin lesions called acrodermatitis chronica atrophicans. If untreated, infection can last for months to years; in mouse studies, organisms can be reisolated from almost any tissue throughout the lifetime of the animal. Despite this pattern of pathogenesis, *B. burgdorferi* produces no known toxins. Lyme disease *Borrelia* thus appear to represent non-toxigenic, invasive, persistent pathogens that cause disease through the induction of host inflammatory reactions, similar to *Treponema pallidum* (syphilis) and *Mycobacterium tuberculosis* (Norris et al., 2010).

Since its initial discovery and culture in 1981, *B. burgdorferi* has been the subject of intensive study in an attempt to better understand the biology of this organism and thereby identify properties useful in the diagnosis, treatment, or prevention of Lyme disease. This work has resulted in tremendous progress (Rosa et al., 2005; Samuels and Radolf, 2010), particularly in terms of understanding the spirochete's molecular biology and the massive gene regulation that accompanies the transition between the disparate mammalian and tick host environments. Despite these advances, several barriers (Table 1) have hampered the ability to fulfill molecular Koch's postulates regarding the role of borrelial genes in biological processes and pathogenesis. While some of these barriers have been at least partially overcome, transformation of low-passage, infectious *B. burgdorferi* remains a challenge. Thus, site-directed mutagenesis of a particular gene may require 3–6 person-months for the transformation process, outgrowth of transformants, screening for mutants with appropriate insertions, and plasmid analysis. As a result, fewer than 100 of the 1739 open reading frames (ORFs) in infectious *B. burgdorferi* have been subjected to site-directed mutagenesis despite intensive efforts by several laboratories.

**Table 1:** **Challenges to genetic manipulation of *Borrelia* species**.

**Barrier**	**Solution(s)**
Fragility of organisms	Specialized methods for preparation of *Borrelia* for electroporation (Samuels, 1995)
Slow growth rate; inefficient isolation of mutants	Utilization of either plating or limiting dilution approaches
Plasmid loss during *in vitro* culture	Minimization of *in vitro* passages (Barbour, 1988)
	Careful monitoring of plasmid content
	Use of shuttle vectors to replace virulence-associated plasmid-encoded genes (e.g., *pncA*)
Restriction-modification (R-M) systems	Disruption of R-M genes
	Use of strains lacking plasmids (lp25, lp56) that encode R-M systems
	DNA methylation prior to transformation

## Transposon mutagenesis of *B. burgdorferi*

**Figure 1:**
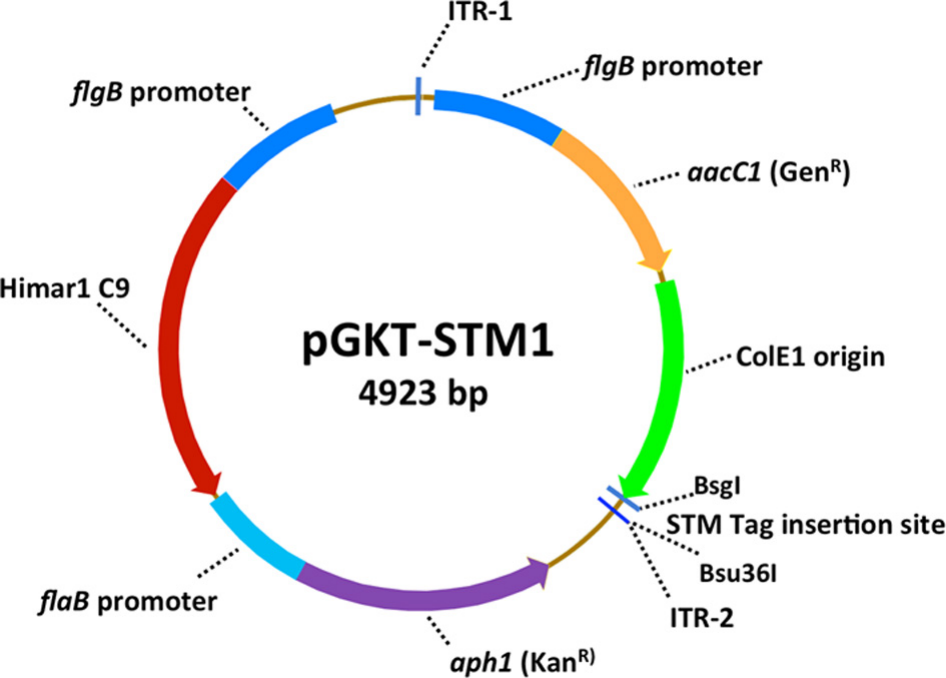
**Diagram of the transposon vector pGKT-STM1, modified from the *Borrelia*-enhanced transposon deliver suicide vector pGKT of Stewart et al. (Stewart and Rosa, 2008)**. Features of pGKT include the use of *B. burgdorferi flgB* and *flaA* promoters to increase the efficiency of expression of the Himar1 c9 transposase and the gentamicin and kanamycin antibiotic resistance markers. The transposable element is flanked by the inverted tandem repeats ITR-1 and ITR-2, and contains the *aacC1* gentamicin resistance cassette and the ColE1 origin of replication (to permit plasmid replication in *E. coli* and transposon insertion site rescue) (Stewart et al., 2004). The modification in pGKT-STM1 and other STM derivatives is the addition of a unique 7-bp signature tag close to ITR2.

Transposon mutagenesis is a powerful means of producing randomized gene mutations in bacterial genomes (Beaurepaire and Chaconas, 2007). In 2004, Stewart et al. (2004) reported the development of a *Himar1*-based transposon suicide vector called pMarGent for use in *Borrelia* organisms. *Himar1* is a transposon of the *mariner* family that was originally isolated from the blowfly, *Drosophila mauritiana*. Lampe et al. (1999) selected point mutants of the *Himar1* transposase (included so-called C9 and A7 derivatives) that exhibited increased transposition rates; the C9 variant was used in pMarGent. A modified version of pMarGent called pGKT (Figure 1) was later developed to include a second selectable marker (Kan^R^) in the non-transposed “backbone” in addition to the gentamycin resistance gene present in the transposable element (Stewart and Rosa, 2008). This modification greatly increases the stability of the vector in *E. coli* and facilitates additional alterations (such as the addition of signature tags). In both pMarGent and pGKT, the transposable element consists of the *B. burgdorferi* constitutive promoter *flgB_P_* coupled with the gentamicin resistance cassette *aacC1* and the ColE1 origin of replication flanked by two *Himar1* inverted tandem repeat sequences (Figure 1). The non-transposed region of pGKT includes *flgB_P_*::C9 transposase gene and the *B. burgdorferi flaB* promoter with the *aph* kanamycin resistance cassette. *mariner*-based transposons insert at any 5′-TA-3′ sequence with no apparent sequence bias, and result in duplication of the TA sequence at the other end of the transposon. Stewart et al. (2004) demonstrated the apparent random insertion of the pMarGent transposable element in both the chromosome and plasmids with high transformation efficiency (>3 × 10^−5^) in the non-infectious *B. burgdorferi* clones B31-A*chb*C72 and A3-89. However, these strains lack both lp25 and lp56, which contain the restriction-modification genes *bbe02* and *bbq67* (Lawrenz et al., 2002); lp25 also contains the *pncA* nicotinamidase gene that is required for mammalian and tick infection (Purser et al., 2003; Deneke and Chaconas, 2008). Attempts to transform the low-passage, infectious *B. burgdorferi* A3 and N40 strains (which contain lp25) with pMarGent were unsuccessful (Stewart et al., 2004).

In 2004, Kawabata et al. (2004) introduced the infectious, transformable *B. burgdorferi* strains 5A4NP1 and 5A18NP1. Both strains have a partial deletion and insertion of an *aph* Kan^R^ cassette in *bbe02*; 5A18NP1 also lacks the plasmid lp56 and hence *bbq67*. In electroporation studies with a shuttle vector, 5A4NP1 and 5A18NP1 yielded 10 colonies/μg DNA and 600 colonies/μg DNA, respectively; in contrast, the parental strains 5A4 and 5A18 (which contain functional *bbe02*) had <1 and 14 colonies per μg DNA. These strains thus reduce the transformation barrier while retaining full infectivity in the mouse model (Kawabata et al., 2004).

Utilization of pMarGent in combination with strain 5A18NP1 resulted in the first successful transposon mutagenesis of infectious *B. burgdorferi* (Botkin et al., 2006). A small library of 33 mutants was examined for transposon insertion site by *E. coli* rescue (Stewart et al., 2004), plasmid content, and infectivity in C3H/HeN mice. Mutations in the genes encoding IMP dehydrogenase (GuaB, involved in inosine-guanine interconversion) and the flagellar switch protein FlaG-1 were found to render *B. burgdorferi* non-infectious, but complementation was not attempted in these experiments. This study, although limited, indicated the feasibility of larger scale transposon mutagenesis studies.

## Ordered transposon mutant library

In 2007, Lin et al. (2012) began the process of accumulating a comprehensive transposon mutagenesis library utilizing signature-tagged versions of pKGT and the *B. burgdorferi* B31 derivative 5A18NP1. pKGT was modified to contain 12 different 7 bp “signature tags” that could be utilized to distinguish between co-infecting strains. This approach was based on earlier STM studies originated by Holden and colleagues (Hensel et al., 1995), as has been widely used for the study of bacterial-host interactions and pathogenesis. Relatively few signature tags were utilized in the construction of the *B. burgdorferi* library because of the inherent difficulties in producing large numbers of mutants in this organism. Each transformation typically yielded between 200 and 300 transformants, so several years was required to accumulate and characterize the number of clones estimated to yield a near saturation library. Overall, 6325 clones were isolated, and the insertion sites for 4479 clones were mapped by *E. coli* rescue and sequencing; the insertion sites for some transformations were not determined because of a high proportion of sibling clones. The plasmid content of the sequence-defined clones was determined using a novel, multiplex Luminex-based approach in which the samples can be analyzed in a high-throughput, 96-well format (Norris et al., 2011).

The large number of transposon mutant clones isolated precluded the use of re-cloning to assure clone purity. Although only well-isolated colonies were selected, it has been found that a fair number of the preparations contain two co-isolated clones. Colonies of *B. burgdorferi* are diffuse and transluscent, so this co-isolation is most likely due to the presence of overlapping colonies. It is recommended that the transposon mutants be re-cloned prior to detailed analysis and examined for the presence of other clones. The method currently in use involves PCR using primers flanking the transposon insertion site. A “wild type” PCR product the same size as obtained with 5A18NP1 parental strain in addition to a larger product (consistent with the expected transposon insertion) indicates the presence of clone(s) with insertions at other sites.

Each transposition event represents a mini-experiment, in that clones containing transposon insertions that significantly reduce fitness for *in vitro* growth will not survive. The distribution of transposon insertion sites thus provides a map of the genes and other elements essential for *in vitro* culture or replicon maintenance. The map is incomplete in the case of the existing *B. burgdorferi* transposon mutagenesis library, in that the library is not saturating. However, patterns can be discerned based on the lack of crippling mutations in groups of required genes. For example, the only ribosomal protein gene mutation isolated was in the last 10% of the ORF for the S21 protein, consistent with this mutation resulting a truncated but functional product. The 22 clones recovered with mutations in one of the two 23S ribosomal RNA genes had no obvious growth defect, indicating that cells are functional with only a single copy of this redundant gene. Only 34% of predicted protein-encoding genes in the chromosome had insertions, whereas 57% of predicted plasmid genes had insertions, most likely because of the higher proportion of essential genes required for *in vitro* culture in the chromosome. Variability occurred in the “hit rate” of both genes and replicons, with some genes and plasmids having as high as 23.06 (conserved hypothetical gene *bb0017*) and 18.01 (circular plasmid cp9) unique insertions per kb of DNA, respectively. The reason for this variability is not known, but conceivably may be related to improved *in vitro* growth characteristics in some mutants. Core gene classes that did not have debilitating mutations included those involved in DNA synthesis, transcription, translation, protein translocation, peptidoglycan synthesis, glycolysis, and pentose phosphate pathway, lipid interconversion, the V-type ATPase (presumably involved in maintaining the proton gradient across the cytoplasmic membrane), and the ubiquitous chaperones GroE and HP70 (Lin et al., 2012). Gene mutations that were permissive for *in vitro* growth included most of the numerous lipoprotein genes and several genes involved in chemotaxis. Whereas an intact chemotaxis apparatus is not required for culture, relatively few mutants were obtained in flagellar genes. Those mutants that were obtained in flagellar genes often produced elongated cells, indicative of defective cell division. Thus, spirochetal motility may facilitate the cell division process.

## Detection of virulence determinants in functional groups

Screening of mutants for mouse infectivity by needle inoculation has been initiated using a Luminex-based STM analysis approach (Lin et al., 2012). In a typical STM experiment, 11 mutants, each with a different signature tag, were inoculated subcutaneously at 10^5^ of each mutant into six C3H/HeN mice. Groups of three mice were analyzed at 2 and 4 weeks post-inoculation (PI), and up to five tissues (joint, heart, bladder, inoculation site, and a distal skin site) were examined from each mouse. In many experiments, the tissue specimens were each examined by both direct extraction of DNA from the tissue and by culture of the *B. burgdorferi* from the tissue followed by DNA extraction from the culture. A semi-quantitative, high-throughput Luminex© FlexMap™ procedure was developed for detection of *B. burgdorferi* STM mutants in mammalian hosts. The Luminex technology allows the assessment of up to 200 signals in a single well of a 96-well plate, thus providing a high-throughput method. Five tissues per mouse (joint, heart, bladder, inoculation site, and a distal skin site) were examined at two time points (2 and 4 weeks) with three mice each. A relatively small number of signature-tagged mutants were utilized in each STM infection experiment because of the challenges of constructing *B. burgdorferi* mutant libraries with multiple sequence tags, as well as the potential of a bottleneck effect. By combining the multiple mice, time points, tissues, and DNA preparation procedures, up to 60 data points per clone were obtained in a typical STM experiment (Lin et al., 2012). The data consisted of the Median Fluorescence Intensity (MFI) values obtained by Luminex analysis for each clone. Results obtained with representative infectious and non-infectious clones indicated that MFI values <100 (arbitrary units) were consistent with low infectivity. Variability in the numbers of organisms in individual tissue specimens occurs because of the paucibacillary nature of infection, and averaging of the multiple data points was found to yield more reliable results.

Functional genomics and pathogenomics approaches were applied to select mutants for the analysis of the roles of *B. burgdorferi* genes in metabolism, structure, pathogenesis, and other biological functions. The infectivity of 502 mutants in 422 genes has been tested (Lin et al., 2012), and the roles of some of these genes in pathogenesis and biological activities are being characterized more fully. Transposon mutations in genes associated with DNA recombination and repair, chemotaxis, motility, transport systems, plasmid maintenance, genes involved in gene regulation, sRNA genes, metabolic pathways, proteases, complement regulator-acquiring surface proteins (CRASPs), predicted lipoproteins, conserved hypothetical proteins (CHPs), and hypothetical proteins (HPs) of *B. burgdorferi* were selected for STM screening. A representative number of mutants in intergenic regions were also tested for infectivity to evaluate potential polar effects, small RNA genes, and regulatory regions (Lin et al., 2012). Some of the mutants exhibiting reduced infectivity in the STM screen are listed in Table 2. As an example, the effects of mutations in 23 genes involved in DNA recombination and repair on *vlsE* recombination were determined, and the results indicated that transposon mutants in *ruvA, ruvB*, and *mutS* exhibited reduced infectivity in mice and diminished *vlsE* recombination. Using this approach and site-directed mutagenesis, RuvA, RuvB, and MutS were the first trans-acting factors identified as necessary for *vlsE* recombination and antigenic variation (Dresser et al., 2009; Lin et al., 2009).

**Table 2:** ***B. burgdorferi* B31 virulence determinant candidates as indicated by STM analysis^a^**.

**Functional categories and gene products**	**Genetic locus**	**STM mouse infectivity phenotype**	**Verification by needle (N) or tick (T) inoculation (References)**
**DNA RECOMBINATION AND REPAIR**			
RuvA	BB0023	Intermediate infectivity	N (Lin et al., 2009); T (Lin et al., 2009)
RuvB	BB0022	Intermediate infectivity	N (Lin et al., 2009); T (Lin et al., 2009)
MutS	BB0797	Intermediate infectivity	N (Lin et al., 2009)
**CHEMOTAXIS**			
CheA1	BB0567	Non-infectious	T (Lin et al., 2012)
CheA2	BB0669	Non-infectious	T (Lin et al., 2012)
CheB1	BB0415	Intermediate infectivity	
CheB2	BB0568	Non-infectious	T (Lin et al., 2012)
CheR2	BB0414	Intermediate infectivity	
CheW2	BB0565	Non-infectious	
CheW3	BB0670	Non-infectious	
CheX	BB0671	Non-infectious	
CheY2	BB0570	Non-infectious	
SulP	BB0566	Non-infectious	
Mcp1	BB0578	Non-infectious	T (Lin et al., 2012) (intermediate)
Mcp3	BB0597	Non-infectious	
Mcp4	BB0680	Non-infectious	T (Lin et al., 2012)
Mcp5	BB0681	Non-infectious	T (Lin et al., 2012)
**FLAGELLAR STRUCTURE AND ASSEMBLY**			
FliG1	BB0221	Non-infectious	
FliZ	BB0276	Non-infectious	
FlbA	BB0287	Non-infectious	T (Lin et al., 2012)
FliI	BB0288	Non-infectious	T (Lin et al., 2012)
FliH	BB0289	Non-infectious	T (Lin et al., 2012)
FlaA	BB0668	Non-infectious	T (Lin et al., 2012)
FlgI	BB0772	Non-infectious	
**PHOSPHOTRANSFERASE SYSTEM**			
ptsG	BB0645	Non-infectious	
FruA1	BB0408	Non-infectious	
FruA2	BB0629	Variable infectivity	
MalX1	BB0116	Intermediate infectivity	
MalX2	BBB29	Intermediate infectivity	
ChbB	BBB06	Non-infectious	
**ABC TRANSPORT PROTEINS, *BORRELIA* EFFLUX SYSTEM (Bes), AND OTHER TRANSPORT SYSTEMS**			
ProX	BB0144	Intermediate infectivity	
MglA	BB0318	Non-infectious	
ABC transporter ATP-binding protein	BB0573	Non-infectious	
OppA-2	BB0329	Intermediate infectivity	
OppA-3	BB0330	Intermediate infectivity	
OppA-4	BBB16	Variable infectivity	
OppA-5	BBA34	Variable infectivity	
BesA	BB0141	Intermediate infectivity	
BesC	BB0142	Intermediate infectivity	
LctP	BB0604	Non-infectious	
NhaC-1	BB0637	Non-infectious	
NhaC-2	BB0638	Non-infectious	
Na^+^/Ca^+^ exchange protein	BB0164	Intermediate infectivity	
GlpF	BB0240	Intermediate infectivity	
GltP	BB0729	Intermediate infectivity	
purine permease P1	BBB22	Intermediate infectivity	

a *Transposon mutants lacking infectivity-related plasmids (lp25, lp28-1, lp36) or with insertions in the last 10% of the gene were excluded from this analysis. Non-infectious, mean MFI <100 and <20% of tissue sites with MFI >100. Intermediate infectivity, mean MFI between 100–500, and 20–50 percent of tissue sites with MFI >100. Variable infectivity, independent transposon mutants yielded different infectivity results. Transposon mutant STM infectivity results that have been verified by needle or tick inoculation with individual clones are shown with the pertinent reference(s)*.

Motility and chemotaxis have long been thought to be key factors in borrelial infectivity and pathogenesis, but genetic evidence for that role has been obtained only recently (Li et al., 2010; Sze et al., 2012; Guyard et al., 2013; Sultan et al., 2013). In the STM transposon mutant analysis, 10 of 14 mutations in chemotaxis genes resulted in a severe loss of infectivity, indicating that the chemotaxis pathway is critical to infectivity (Lin et al., 2012). The genes involved in flagellar structure and assembly are also required for mouse infection. Some of these mutants, such as *fliH, fliI*, and *flbA*, had reduced motility, division defects, and structural changes in the flagellar motor. The cell division, motility, and structural defects of these mutants may all play a role in the observed low infectivity phenotype.

Transport is another key function in pathogenesis. The phosphoenolpyruvate phosphotransferase system (PEP-PTS) plays an important role in carbohydrate transportation and phosphorylation, also a potential role in gene regulation. STM results consistent with non-infectivity were observed for a transposon mutant in the gene encoding the glucose-glucoside specific IIBC component PtsG (Table 2); reduced infectivity was observed with mutants in the genes for the maltose specific PTS transporters MalX-1 and MalX-2 and the fructose-mannose transporter FruA-1. Disparate infectivity results were obtained with two mutants in the gene encoding the fructose-mannose specific IIB component FruA-2, so further analysis is needed. The Chb system is thought to play important roles in the utilization of chitobiose during infection of ticks (Tilly et al., 2001; Rhodes et al., 2010; Sze et al., 2013). Not surprisingly, inactivation of the genes encoding the chitobiose-specific components ChbA and ChbC did not affect mouse infection by needle inoculation in our system. However, the chitobiose-specific IIBC component ChbB mutant tested exhibited a no-infectivity phenotype (Lin et al., 2012); this finding will require further verification and investigation. In addition, the ABC transporter genes encoding the ATP-binding protein of the galactose transporter MglA, ProX, and an uncharacterized ATP-binding protein (*bb0573*) exhibited a low infectivity phenotype in the STM system. The five OppA oligopeptide ABC periplasmic binding proteins of *B. burgdorferi* exhibit varied infectivity phenotypes, but obviously have required specific binding properties. Genes encoding the lactose permease (LctP), Na^+^/H^+^ antiporter proteins (NhaC-1 and NhaC-2) also appear to required for mouse infection. Mutation of *Borrelia* efflux system genes *besC* and *besA*, Na^+^/Ca^+^ exchange protein (*bb0164*), glycerol uptake facilitator GlpF (*bb0240*), glutamate transporter (*bb0729*), and purine permease P1 (*bbB22*) resulted an intermediate infectivity phenotype (Table 2).

## Screening of virulence genes in infectivity-related plasmids

Lyme disease *Borrelia* species typically contain over 20 linear and circular plasmids. These replicons have been called “minichromosomes” because several are required for the complex life cycle of these organisms. *Himar1*-based transposon mutagenesis resulted in over 2500 sequence-defined unique insertion sites in the *B. burgdorferi* plasmids, disrupting 503 of 790 ORFs (68%); the proportion of ORFs disrupted in each plasmid ranged from 29 (in lp5) to 91% (cp9). A high proportion of borrelial plasmid genes do not have orthologs in other organisms, so many have no predicted function. There are also many paralogous families among the plasmid genes. Although over 30 *B. burgdorferi* plasmid genes have been successfully inactivated by site-directed mutagenesis (Rosa et al., 2005), the availability of the transposon mutant library will yield valuable information about these enigmatic replicons and their genes.

**Figure 2:**
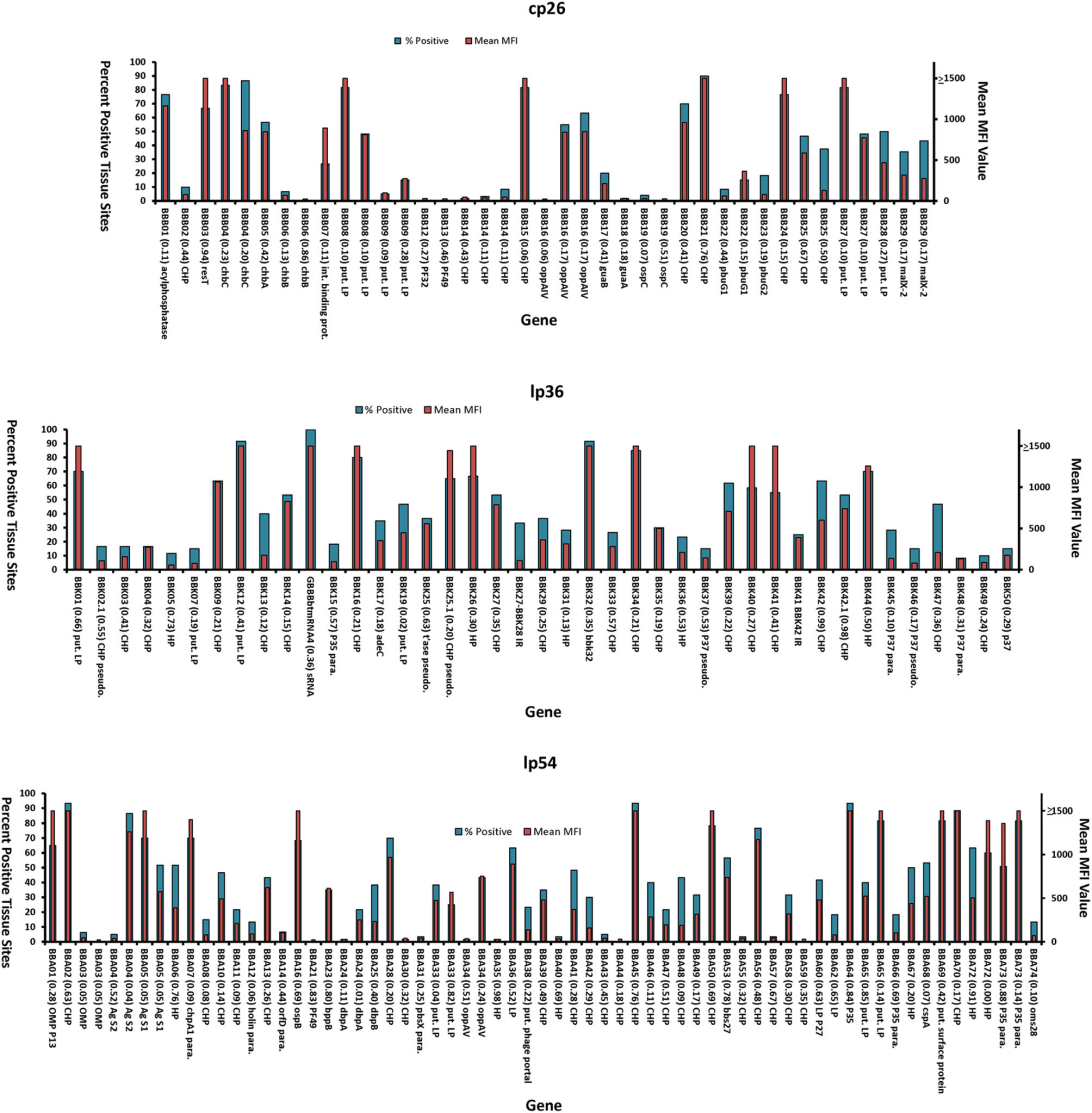
**Mouse infectivity of transposon mutants in plasmid-encoded genes, as determined by Luminex-based STM analysis**. Results obtained for genes in plasmids cp26, lp36, and lp54 are shown. For each mutant, the data represent the percent of positive tissue sites (MFI >100; blue bars) and mean MFI value (red bars) for 30–60 data points. Only clones that have all plasmids known to be required for mouse infection (cp26, lp25, lp28–1, lp36, and lp54) were analyzed. The gene designation, insertion ratio (bp from 5′ end to insertion/total bp), and description are provided. Abbreviations: HP, hypothetical protein; CHP, conserved hypothetical protein; para., paralog; pseudo., pseudogene; put. LP, putative lipoprotein; t'ase, transposase.

To date, representative mutants in genes associated with the infectivity-related plasmids cp26, lp25, lp28-1, lp36, and lp54 were analyzed using the STM process and Luminex-based technology (Lin et al., 2012). A summary of the results obtained for cp26, lp36, and lp54 are displayed in Figure 2. The percentage of positive tissue sites and mean MFI value were found to be useful parameters in summarizing the data from many animals, time points, tissue sites, and sampling methods, resulting in up to 60 data points. These two criteria yielded comparable patterns (Figure 2). The contiguous range of values sometimes does not permit a clear “±” result, resulting in an intermediate (or indeterminate) infectivity category in some instances. Nevertheless, the results obtained with mutants in these plasmids provide evidence that a high number of genes encoded in these plasmids appear to be required for full infectivity in mice. STM analysis is considered a screening procedure, and all results obtained by this method need to be confirmed by single clone inoculation and genetic complementation.

Transposon insertions were obtained in 26 of the 29 genes in cp26. Inactivation of 11 genes appeared to render a non-infectious phenotype based on the STM analysis, including those encoding chitobiose transporter ChbB, the PF32 and PF49 plasmid maintenance proteins, CHP BBB14, GMP synthase GuaA, and OspC (Figure 2). In addition, mutations in the genes encoding the CHP BBB02, HP gene BBB09, GuaB (inosine-5-monophosphate dehydrogenase, and purine permeases PbuG1 and PbuG2) yielded reduced STM parameter values (Figure 2) (Lin et al., 2012). Mutants in the genes encoding OspC, GuaA, GuaB, PbuG1, and PbuG2 had all been identified as virulence determinants previously (Byram et al., 2004; Grimm et al., 2004; Pal et al., 2004; Stewart et al., 2006; Tilly et al., 2006, 2009; Jewett et al., 2007a).

Of the 60 annotated genes in lp36, 38 protein-encoding genes have been disrupted. Transposon insertions were also found in one small RNA gene and two intergenic spacers. A total of 41 unique mutants have been examined for their infectivity in STM system (Figure 2). Consistent with previously published report by Jewett et al. (2007b), our result indicates that inactivation of the gene encoding adenine deaminase AdeC resulted in reduced mouse infection. Disruption of the BBK32 fibronectin-binding protein gene did not have an apparent effect on infectivity. BBK32 is involved in vascular endothelium adherence and dissemination, but is not absolutely required for infection (Li et al., 2006; Seshu et al., 2006; Norman et al., 2008; Moriarty et al., 2012); it is possible that the high infectious dose (10^5^) used for each mutant in the STM analysis was sufficient to establish disseminated infection. Mutations in a region encompassing *bbk02.1* to *bbk07* (a poorly defined region in which some of the genes are no longer annotated) resulted in decreased infectivity. Mutations in several other CHP genes appear to result in reduced infectivity, including those in *bbk15*, and putative lipoprotein genes *bbk13, bbk36, bbk37*, and *bbk45* through *bbk50* (Figure 2) (Lin et al., 2012). *In vivo* expression technology (IVET) studies by Ellis et al. (2013) indicated that *bbk46* is highly expressed during mouse infection, and further analysis showed that a *bbk46* mutant was defective in persistent infection of mice.

Linear plasmid lp54 is one of the most highly conserved elements in the *Borrelia* genome. A total of 75 protein-encoding genes are encoded on lp54, and some of them are differentially expressed in tick vector and mammalian hosts. Most proteins encoded on lp54 do not have homologs in other organisms and are of unknown function (Casjens et al., 2000; Jewett et al., 2007a). Transposon mutants were obtained in 58 genes on lp54, and a high proportion of these mutants had decreased infectivity (Figure 2). Most of these are *Borrelia* species-specific proteins with unknown functions and several are *B. burgdorferi*-specific proteins. Transposon insertions in BBA03 (outer surface protein), BBA14 (OrfD paralog), BBA21 (PF49), BBA24 (DbpA), BBA30 (CHP), BBA31 (CHP), BBA35 (HP), BBA40 (HP), BBA43 (CHP), BBA44 (CHP), BBA55 (CHP), BBA57 (CHP), and BBA59 (CHP) resulted in a non-infectious phenotype by STM analysis. Additional mutants exhibited reduced STM analysis parameters, and may also have attenuated phenotypes. Disparate results were obtained for *bba04*, with two clones having high and low infectivity phenotypes (Figure 2) (Lin et al., 2012). Bestor et al. (2012) determined previously that a *bba03* deletion mutant had a competitive disadvantage in tick-mediated infection of mice in comparison to the wild type parental strain; a deletion mutant lacking *bba01-bba07* was further attenuated, indicating that other genes in this region may be required for full infectivity in mice. In other studies, site-directed disruption of the decorin binding protein DbpA was found to reduce mouse infectivity by needle inoculation but not by tick-mediated infection (Hagman et al., 2000); the transposon mutant results are consistent with the prior needle inoculation findings. Mutation in gene of *ospB* had little apparent effect on mouse infection, although this surface lipoprotein expressed predominantly in the tick infection and play the important role in tick midgut colonization. BBA64 is a member of the P35 paralogous protein family, and previous studies indicated that BBA64 is required for tick transmission (Gilmore et al., 2010), but mutants in this gene had only minor effects on infection by needle inoculation (Maruskova and Seshu, 2008; Maruskova et al., 2008). The STM analysis was consistent with the latter result, in that the available *bba64* mutant (which had a transposon insertion in last 16% of reading frame) was fully infectious (Figure 2); in this case, however, it is possible that the mutant expresses a truncated but functional protein. Further studies with BBA64 immunization indicate that antibodies against this protein do not protect against needle or tick infection (Brandt et al., 2014). STM analysis indicated that mutation of lp54-encoded protein Oms28 (*bba74*) resulted in decreased infectivity (Lin et al., 2012), but these results are not consistent with prior studies with B31 A3, which apparently expresses a truncated BBA74 product (Mulay et al., 2009).

BptA, PncA, and Bbe31 encoded on lp25 had been identified as virulence determinants previously (Purser et al., 2003; Revel et al., 2005; Strother et al., 2005; Zhang et al., 2012). The transposon mutant library contains insertions in the genes encoding BptA and PncA, and these mutants exhibited reduced or non-infectivity as reported previously. In addition, mutations in genes of *bbe04.1, bbe09, bbe18, bbe19, bbe24*, and *bbe29.1* substantially reduced infectivity, and intermediate infectivity was observed in mutants in genes of *bbe07* and *bbe18* (Lin et al., 2012).

In the STM studies, low infectivity was observed among lp28-1 mutants of genes encoding BBF03, BBF05, BBF10, BBF18, and BBF25. Mutation of genes of BBF04, BBF07, BBF08, BBF12, BBF19 appear to cause reduce infectivity. Interestingly, transposon insertions in intergenic spacers between *bbf25-26* and *bbf28-29* causes severe defect in spirochete infectivity (Lin et al., 2012). The *vls* locus involved in VlsE antigenic variation and immune evasion is located at one end of lp28-1 (Zhang et al., 1997). Only one transposon insertion was obtained in the *vls* silent cassettes, and none were obtained in the *vlsE* expression site (Lin et al., 2012). The silent cassette mutant was infectious, indicating that interruption of the silent cassette region is not sufficient to inhibit the *vlsE* recombination process.

## Tn-seq

Transposon insertion site sequencing approaches such as Tn-seq is a powerful genetic screening technique in which high-throughput sequencing is used as a detection method in transposon mutagenesis screens to identify the contribution of individual genes to bacterial fitness under specific growth conditions (van Opijnen and Camilli, 2013). A library of transposon mutants (input pool) can be utilized to infect animals or subjected to *in vitro* selection conditions, and the resulting organisms (output pool) collected. Amplification of the transposon insertion site region and massively parallel sequencing provides a high-throughput comparison of the input pool and output pool. In comparison with STM approaches, Tn-seq has several advantages (Table 3), including the ability to examine an entire library in one set of replicate experiments without prior characterization of the insertion sites. For *Borrelia* studies, a disadvantage is that the plasmid content of each clone is not known, so the low infectivity of a particular mutant may be due to concomitant plasmid loss. Thus, presence of more than one clone with a mutation in a particular gene in the input pool and the underrepresentation of those clones in the output pool may be necessary to establish the gene product as a potential virulence determinant.

**Table 3:** **Advantages and disadvantages of ordered library, signature-tagged mutagenesis (STM) and Tn-seq approaches**.

**Approach**	**Advantages**	**Disadvantages**
STM analysis	Does not require availability of high-throughput sequencing	Requires sequence analysis of the Tn insertion points of individual clones of interest either before (ordered library) or after STM screening
	Tn mutant clones available for further analysis after STM screening	Relatively low throughput method that requires multiple animal experiments for infectivity screening
	Relatively small number of clones used per experiment decreases possibility of bottleneck effects	Potential cross-contamination during clone isolation can complicate interpretation
Tn-seq	Capacity to screen an entire library in a single infectivity experiment	Lack of plasmid content information; low infectivity may be related to plasmid loss
	Does not require isolation and characterization of individual Tn mutant clones	Tn mutants of interest would have to be re-isolated for further study (if not from an ordered library)
	Easier application to *in vitro* screening methods (e.g., metabolic, adherence, or cell co-culture experiments)	Possible bottleneck effects (non-uniform recovery of organisms) may necessitate use of large numbers of animals or cultures
		Relatively high minimum analysis cost

In 2013, Troy et al. adapted Tn-seq for use in studies of *B. burgdorferi* using the STM transposon library (Troy et al., 2013). In a Tn-seq experiment, the transposon library is subjected to growth under a test condition. Genomic DNA is then isolated from the *B. burgdorferi* population before and after selection. The DNA is fragmented and cytosine tails (C-tails) are added using terminal deoxynucleotidyl transferase. The addition of a C-tail allows for the amplification of the transposon-genomic DNA junction using primers specific to the ColE1 site on the end of the transposon and the C-tail. The relative abundance of each mutant is determined by sequencing the chromosomal DNA flanking the transposon *en masse* using a primer that anneals to the end of the transposon. In this way, the exact location of every transposon insertion present in the library is revealed. The frequency of the insertion sequence in the library corresponds to the frequency of the transposon mutant containing that insertion in the original bacterial populations. The fitness of each insertion mutant in the test condition is determined by comparing the relative frequency of each mutant before and after growth under the selective pressure. Sequences that decrease in frequency in the library after selection have insertions in genes contributing to bacterial growth in the test condition. The quantitative nature of the high-throughput sequencing enables the discovery of unknown growth determinants including the differentiation of factors with partial phenotypes and/or those acting in redundant pathways. Furthermore, due to the non-specific nature of the genome library preparation, this Tn-seq technique can be used with any transposon as long as the sequence of the 3′ end of the transposon is known.

Tn-seq is particularly amenable to *in vitro* screens as unlike STM experiments that are limited to a small subset of mutants in each experiment, the entire transposon library can be tested in a single competition assay. Tn-seq has also been used to screen *in vivo* fitness. However, a potential complication of animal screens is a population bottleneck at the site of infection that results in the stochastic loss of a significant portion of the injected *B. burgdorferi* by 3 days post-infection. If all transposon mutants containing a particular insertion are lost at the inoculation site due to the bottleneck rather than decreased fitness, the disrupted gene may be misidentified as contributing to bacterial survival during infection. However, experiments by Troy et al. have demonstrated that the effects of this bottleneck can be circumvented, at least in part, by combining results from the *B. burgdorferi* populations recovered from multiple mice infected with the same inoculum (Troy et al., 2013). Part of the bottleneck effect is due to innate immune mechanisms (resulting in a non-specific reduction of the inoculated population), as demonstrated by the reduced magnitude of the bottleneck in MyD88^−/−^ mice in comparison with wild type mice.

## Conclusions

Results obtained to date with *B. burgdorferi* transposon mutants indicate that a high proportion of genes are required for mouse infection. The assessment of multiple tissue sites at two different time points using Luminex FlexMap technology provides a robust data set. Tn-seq has multiple advantages and is expected to greatly accelerate the application of transposon mutant libraries to studies of infectivity and other biological processes. Both STM and Tn-seq are considered screening methods, so the results must be confirmed by studies with isolated transposon mutants (or independently derived mutants) and genetic complemention. In addition, polar effects on downstream genes should be verified for observed reduced infectivity phenotype. All mutants should be re-cloned prior to more thorough studies due to the potential occurrence of co-isolation of two or more clones in a single colony. Finally, cross-complementation may occur between clones in the transposon library. We believe that the continued analysis of the mutant library will lead to a more comprehensive delineation of the virulence determinants of *B. burgdorferi* and will improve our understanding of the novel pathogenic and biologic properties of Lyme *Borrelia* organisms and other invasive, non-toxigenic, persistent pathogens.

### Conflict of interest statement

The authors declare that the research was conducted in the absence of any commercial or financial relationships that could be construed as a potential conflict of interest.
